# Functional and Therapeutic Potential of *Cynara scolymus* in Health Benefits

**DOI:** 10.3390/nu16060872

**Published:** 2024-03-17

**Authors:** Chiara Porro, Tarek Benameur, Antonia Cianciulli, Mirco Vacca, Margherita Chiarini, Maria De Angelis, Maria Antonietta Panaro

**Affiliations:** 1 Department of Clinical and Experimental Medicine, University of Foggia, 71122 Foggia, Italy; chiara.porro@unifg.it; 2 Department of Biomedical Sciences, College of Medicine, King Faisal University, Al-Ahsa 31982, Saudi Arabia; tbenameur@kfu.edu.sa; 3 Department of Biosciences, Biotechnologies and Environment, University of Bari, 70125 Bari, Italy; antonia.cianciulli@uniba.it; 4 Department of Soil, Plant and Food Science, University of Bari Aldo Moro, 70125 Bari, Italy; mirco.vacca@uniba.it (M.V.); maria.deangelis@uniba.it (M.D.A.)

**Keywords:** artichoke, inflammation, functional food, polyphenols

## Abstract

Dietary supplements enriched with bioactive compounds represent a promising approach to influence physiological processes and enhance longevity and overall health. *Cynara cardunculus* var. *scolymus* serves as a functional food supplement with a high concentration of bioactive compounds, which offers various health-promoting benefits. Several chronic diseases have metabolic, genetic, or inflammatory origins, which are frequently interconnected. Pharmacological treatments, although effective, often result in undesirable side effects. In this context, preventive approaches are gaining increased attention. Recent literature indicates that the consumption of bioactive compounds in the diet can positively influence the organism’s biological functions. Polyphenols, well-known for their health benefits, are widely recognized as valuable compounds in preventing/combating various pathologies related to lifestyle, metabolism, and aging. The *C. scolymus* belonging to the Asteraceae family, is widely used in the food and herbal medicine fields for its beneficial properties. Although the inflorescences (capitula) of the artichoke are used for food and culinary purposes, preparations based on artichoke leaves can be used as an active ingredient in herbal medicines. *Cynara scolymus* shows potential benefits in different domains. Its nutritional value and health benefits make it a promising candidate for improving overall well-being. *C. scolymus* exhibits anti-inflammatory, antioxidant, liver-protective, bile-expelling, antimicrobial, and lipid-lowering neuroprotective properties. Different studies demonstrate that oxidative stress is the leading cause of the onset and progression of major human health disorders such as cardiovascular, neurological, metabolic, and cancer diseases. The large amount of polyphenol found in *C. scolymus* has an antioxidant activity, enabling it to neutralize free radicals, preventing cellular damage. This reduces the subsequent risk of developing conditions such as cancer, diabetes, and cardiovascular diseases. Additionally, these polyphenols demonstrate anti-inflammatory activity, which is closely associated with their antioxidant properties. As a result, *C. scolymus* has the potential to contribute to the treatment of chronic diseases, including intestinal disorders, cardiovascular diseases, and neurodegenerative pathologies. The current review discussed the nutritional profiles, potential benefits, and pharmacological effects of *C. scolymus.*

## 1. Introduction

Healthy diet and lifestyle are vital for maintaining health and preventing illnesses. In recent years, various research lines have highlighted the correlation between lifestyle choices, such as diet and nutritional factors, and the onset of diseases, especially neurodegenerative ones. In the last few decades, functional food has received great attention.

The term “functional food” encompasses foods or nutrients containing bioactive phytoconstituents that exert beneficial effects on one or more body functions. As such, these functional foods have the potential to enhance health and/or reduce the risk of developing certain diseases when consumed in quantities typical of a regular diet [1].

In this regard, polyphenols belong to a broad class of organic substances that have significant health advantages. The daily diet provides varying amounts of polyphenols, which are concentrated in plant-based products such as fruits, vegetables, spices, nuts, cocoa, tea, and extra virgin olive oil. Some plant sources are particularly rich in polyphenols, which are extracted, concentrated, and marketed for their health benefits [2]. Examples of polyphenol-rich plant extracts include those from artichoke. The artichoke (*Cynara cardunculus* var. *scolymus*), an *Asteraceae* family member, is native to the Mediterranean region (North Africa and southern Europe) and is widely used as a source of food and medicine. The name was derived from “Kynara”, an Aegean Island where it was cultivated first, or from its fertilizer, ashes, or Cineres in Latin. It belongs to the second-largest family in the plant kingdom comprising more than 2000 species, namely, *Asteraceae* family or Thistle family. Italy holds the world record in the production of this vegetable (approximately 30%): the areas of greatest production are Sicilia, Sardinia, and Apulia. The artichoke is called *Cynara cardunculus* L. in Latin. It includes three botanical varieties: *C. cardunculus silvestris* or wild thistle, *C. cardunculus scolymus* or cultivated artichoke, *C. cardunculus altilis* or domestic thistle [3]. The plant represents a potential source of minerals, and it contains a substantial amount of natural antioxidants, such as vitamin C, as well as numerous polyphenols particularly chlorogenic acid (CLA) and cynarin, flavonoids, and their derivatives, which are known for their health benefits [3]. 

The main edible part of the artichoke is the bud encased in leafy green bracts. The part of the plant comprises the immature inflorescences with fleshy leaves (bracts) and receptacle. However, it is important to note that leaves, external bracts, and stems are not suitable for human consumption and are considered waste generated during the industrial processing of artichoke [4]. Regrettably, the inedible part of the artichoke, comprising 80–85% of the plant biomass, results in the loss of essential phytochemical substances found in different parts of the plant. 

Consequently, utilizing by-products from the non-dietary components can be considered as a valuable source of bioactive molecules, including flavonoids, polyphenols, and others, which can contribute to the treatment of chronic diseases such as cancer, cardiovascular diseases, and neurodegenerative pathologies [5]. This review underscores that *C. scolymus* waste stands out as a compelling ingredient with therapeutic potential. While the accumulation of agro-industrial by-products could pose an environmental challenge, it also presents an opportunity to enhance the productivity and valorization of agricultural by-products, through the production of impactful biomolecules for nutritional purposes. The consumption of artichoke, especially its leaves and extract, has demonstrated effectiveness in lowering levels of bad cholesterol in the blood (LDL), thereby helping to prevent cardiovascular diseases. This benefit is attributed to the presence of inulin, a fiber, and various acids. Furthermore, artichoke has the capacity to reduce the level of triglycerides levels. It is also rich in antioxidants that play a crucial role in combatting the harmful effects of free radicals. Notably, artichokes contain CLA, a strong antioxidant with significant implication for preventing cardiovascular and atherosclerotic diseases. In the mid-20th century, Italian scientists isolated cynarin, a compound from artichoke leaves, and the major di-caffeoylquinic acid derivative of artichoke, which appeared to replicate many of the holistic effects of the entire artichoke [6]. Most of the research on the health benefits of artichoke extracts are associated with the antioxidant potential of phenolic contents, including CLA, caffeic acid, cynarine, and flavonoids. The main polyphenols in artichoke are mono- and di-caffeoylquinic acid derivatives [7]. In this regard, it was also reported that artichoke waste extracts could resist hydrogen peroxide insult, suggesting that more research should be carried out to assess their therapeutic potential [7]. These components have widely attracted scientific interest due to their multiple pharmacological properties, including but not limited to antioxidant, anti-inflammatory, antimicrobial, lipid-lowering, and anticancer effects as it has been demonstrated by a recent study [8]. Additionally, studies have reported that aqueous extracts of *C. scolymus* leaves attenuate oxidative stress and lipoprotein dyshomeostasis in rats fed on high-cholesterol diet. The hepatoprotective effects were elucidated by quantifying the markers of oxidative stress, inflammatory cytokines, insulin signaling, and hepatic gene expression related to lipid metabolism [9].

A recent investigation by Ahmed et al. provided evidence that aqueous extracts from the *C. scolymus* leaves have antidiabetic effects in streptozotocin-induced diabetic rats. Moreover, these leaves showed amelioration in liver function, histological and ultrastructural integrity. These improvements were made possible by the reduction in oxidative stress and inflammation, as well as the enhancement of the antioxidant defense system [10].

Similarly, artichoke ethanol extract exhibited significant protective effects against acute alcohol-induced liver injury, resulting from its anti-inflammatory and antioxidant properties [11]. Additionally, scientific observations have demonstrated that artichoke flowers show anti-inflammatory effects on tissue plasminogen activator-induced inflammation and anti-tumor. In fact, artichoke inhibits the growth of melanoma by promoting apoptosis and inhibiting proliferation [8,12].

Furthermore, it has been extensively documented that artichoke extract is very well tolerated by the human body, as no obvious side effects have been observed, even after several months of continuous administration [13]. For these reasons, artichoke might deserve broad prospects for application in the treatment of chronic inflammation-based diseases due to its antioxidant and anti-inflammatory effects. According to recent reports, artichoke pectin and modified pectin fractions have intestinal anti-inflammatory effects in the dextran sulfate sodium model of mice colitis. This indicates that the administration of artichoke pectin has significantly improved inflammatory bowel disease in this model [14].

In the context of intestinal diseases, the vascular barrier, which is essential for maintaining homeostasis, may be damaged and becomes more permeable. This malfunction of the intestine–vascular unit causes the leakage of inflammatory and bacterial mediators. Once the molecules pass through the bloodstream, they can migrate to any organ in the body, including the brain [15]. 

In this review, we provide a comprehensive summary and discuss recent advances in understanding the mechanisms by which *C. scolymus* extracts modulate the inflammatory biomarkers by exerting a protective effect against inflammation and oxidative stress. We also provide insights and perspectives on the use of *C. scolymus* extracts in managing inflammatory processes that occur in certain chronic diseases such as cardiovascular, intestinal, and neurodegenerative disorders. We recommend further investigations to evaluate the effectiveness of artichoke extracts in treating inflammatory diseases, suggesting that the biomolecules found in *C. scolymus* could emerge as valuable ingredients in the development of health products.

## 2. Bioactive Compounds Extracted from *C. scolymus*

**Figure 1:**
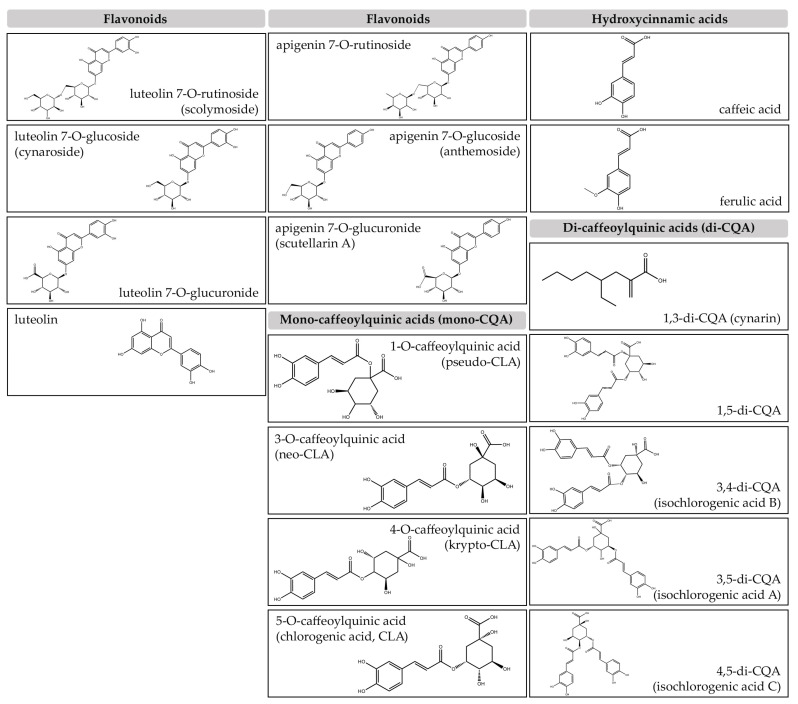
Main compounds featuring artichoke extracts grouped in flavonoids or hydroxycinnamic acids as well as in mono- and di-caffeoylquinic acids, specifically.

The abundant bioactive constituents found in artichoke by-products, including leaves, external bracts and stems, have been extensively documented. These components are characterized by low-fat content and elevated levels of vitamin C, insoluble fibers, inulin, and minerals, such as Potassium (K), Sodium (Na), and Phoshorus (P) [16]. Furthermore, these by-products exhibit notable presence of various phenolic compounds and derivatives, i.e., flavonoids and hydroxycinnamic acids as illustrated in (Figure 1).

The flavonoid category in artichoke includes derivatives of luteolin and apigenin, while its representation of hydroxycinnamic acids is predominantly characterized by caffeic and ferulic acids.

Expanding the spectrum of artichoke’s polyphenolic richness, luteolin-7-O-glucoside, also known as cynaroside, is classified within the flavonoid family.

Flavonoid derivatives are, indeed, pivotal elements found in artichoke extract with anti-inflammatory properties and scavenging activities adding a layer of complexity to the spectrum of benefits linked to artichoke consumption.

Widely present in a variety of plants and fruits, CLA remains the prominent constituent of artichoke extract, particularly representing the most typical form of caffeoylquinic acids. Its widespread recognition is attributable to its antioxidant properties, which significantly contribute to the overall health benefits [7]. Research consistently demonstrates that CLA, through reducing oxidative stress, plays a critical role in enhancing the well-being associated with artichoke consumption. Even cynarin, one of the most important members of this class, is well-known for having possible hepatoprotective effects [5]. As a result, research on its role in promoting liver health has been conducted, making it a focal point in the exploration of benefits of artichoke consumption. While cynarin has been extensively studied by various studies.

According to Schütz et al., cynarin (i.e., 1,3-di-caffeoylquinic acid) is not inherently present in artichoke; rather, it is an artifact formed during aqueous extraction through transesterification of 1,5-di-O-caffeoylquinic acid [17]. Notably, 1,5-di-O-caffeoylquinic acid is recognized as the primary caffeoylquinic acid found in artichoke heads and their pomace [18].

Remarkably, the profile of bioactive components in artichoke extract can be influenced by various factors, including the type of artichoke used, growth conditions, and extraction techniques. As a result, the composition of phenolic compounds in artichoke extracts can display variability. 

In addition to significant bioactive phytochemicals, artichoke contains high concentrations of sesquiterpene lactones, like cynaropicrin, which have a range of biological activities. In this regard, cynaropicrin shows anti-inflammatory, anti-tumor, antioxidative, antiparasitic, antiphotoaging, anti-hyperlipidemic, and anti-inflammatory properties [19,20].

The bioavailability of both polyphenols and sesquiterpene lactones from artichoke was evaluated using Caco-2 cells monolayers as a model of absorption in the large intestine, confirming that artichoke is a nutritionally relevant source of both phenolic compounds and sesquiterpene lactones [21].

The content of sesquiterpene lactones in artichoke leaves varies significantly and depends on the cultivar. Apart from cynaropicrin, dehydrocynaropicrin, and grosheimin, the other two sesquiterpene lactones, cynaratriol and 8-deoxy-11,13-dihydroxygrosheimin, may also be present in comparable quantities [22]. Cynaropicrin, by stimulating the activation of the transcription factor nuclear factor E2-related factor 2 (NRF2), appears to enhance the expression of several antioxidant genes, including glutamate-cysteine ligase and heme oxygenase-1 [23]. NRF2 is considered as the principal regulator of the body’s natural antioxidant defense system. It plays a crucial role in the protection of brain cells from ischemic lesions. Following ischemic damage, the impairment of NRF2 exacerbates the severity of cerebral infarction and aggravates the neurological damage [24]. Furthermore, this natural product has been associated with inhibiting NF-κB. Jin et al. demonstrated that cynaropicrin exhibits anti-inflammatory properties by suppressing the NF-κB pathway [25]. This evidence has been reinforced by other research findings showing that cynaropicrin was able to reduce both the oxidative stress and neuroinflammation in an animal model of ischemic/reperfusion injury in a dose-dependent manner by inhibiting the NF-κB transcriptional activation pathway. NF-κB activation is recognized as an immediate response following the onset of stroke and plays an important role in the disruption of blood-brain barrier, inflammation, and cellular apoptosis. This implies that cynaropicrin has the potential to serve as an effective therapeutic agent for cerebral ischemia-reperfusion injury [25].

Additionally, as reported by Boulos et al. [26], cynaropicrin demonstrates potent inhibition of hematopoietic tumor cells both in vitro and in vivo by inhibiting c-Myc, STAT3, AKT, and ERK1/2, and by suppressing the tubulin network.

Moreover, Matsumoto et al. reported that the methanolic extract of the artichoke leaves inhibited the production of NO in lipopolysaccharide (LPS)-stimulated RAW264.7 cells. Similarly, six different sesquiterpene lactones extracted from artichoke leaves, including cynaropicrin, not only inhibited NO production but also suppressed iNOS induction in LPS-stimulated RAW264.7 cells [27].

This study demonstrated that cynaropicrin inhibited IL-8 and IL-6 mRNA and protein synthesis in LPS-stimulated human gingival fibroblasts in a dose-dependent manner, and that inhibition of *Porphyromonas gingivalis* LPS-induced IL-8 and IL-6 expression by cynaropicrin may be due to NF-κB pathway inhibition. The ability of cynaropicrin to inhibit differentiation of RAW264.7 into osteoclast-like cells was also investigated in the same study, demonstrating that cynaropicrin can significantly reduce RANKL-induced osteoclast differentiation. Furthermore, the anti-inflammatory effects of artichoke extract and cynaropicrin were observed in human gingival fibroblasts, which are key cells in periodontal connective tissues responsible for providing a tissue framework, tooth anchorage, and regulating inflammation. This effect was demonstrated on the expression of inflammatory cytokines induced by LPS in *P. gingivalis.*

This suggests that cynaropicrin appears to be effective in preventing periodontal diseases and could be a valuable molecule in the development of more efficient preventive measures that can protect from periodontal diseases [28].

The Table 1 summarizes the principal mechanisms induced by the artichoke chemical compounds.

## 3. Antimicrobial Activity of Artichoke

The surge in antimicrobial resistance has spurred intensive research into alternative therapeutic agents, and plant-derived compounds have emerged as promising candidates [42]. While artichoke extract has a long history of therapeutic usage, its potential as an antibacterial agent has not been thoroughly investigated, but some studies indicate a wide inhibitory effect against diverse pathogens, indicating a possible application in the food sector [43].

Understanding the synergy and interactions between these constituents is critical for deciphering the mechanisms of artichoke’s antimicrobial effects. Research suggests that artichoke extracts have antimicrobial effects through various mechanisms. Caffeoylquinic acids, for example, have been shown to disrupt bacterial cell walls [44], while flavonoids have been shown to interfere with microbial enzyme activity [45]. These compounds collectively demonstrate a broad spectrum of antimicrobial activity against bacteria, fungi, and even some viruses [46]. The specific molecular targets of artichoke extracts vary, providing a versatile profile for combating diverse pathogens. Numerous studies have investigated the antibacterial potential of artichoke extracts against both Gram-positive and Gram-negative bacteria. Artichoke leaf, head, and stem extracts have shown inhibitory effects on the growth of common pathogens such as *Staphylococcus aureus*, *Escherichia coli*, and *Salmonella* spp. [46].

The ability of artichoke extracts to modulate bacterial virulence factors highlights their potential role in bacterial infection prevention and treatment. Artichoke extracts also have antifungal properties, making them a promising treatment for fungal infections. Various fungi, including *Candida* spp., have been shown to be inhibited in recent research [45]. The interference with fungal cell membrane integrity and the modulation of key enzymes contribute to the antifungal efficacy of artichoke extracts. While the antiviral effects of artichoke extracts have received less attention than the antibacterial and antifungal effects, preliminary research indicates that they may have inhibitory activity against certain viruses. This includes the inhibition of viral replication and the interference with viral attachment to host cells [47]. Nevertheless, further investigations are needed to fully understand the precise mechanisms and potential applications of artichoke extracts in antiviral therapies. This includes exploring potential synergies between artichoke extracts and antibiotics or antifungals to enhance efficacy, reduce required doses, and minimize the risk of developing resistance.

## 4. Inflammatory and Gastrointestinal Diseases and Artichoke

As discussed in various studies, bioactive compounds play a critical role in modulating inflammation, managing, and preventing many inflammatory diseases, including the inflammatory bowel diseases (IBDs) targeting gut microbiota [38,47,48]. As it was already mentioned, artichoke is a widely consumed vegetable known for its potential health benefits. Its function in relation to inflammatory and gastrointestinal (GI) illnesses is the focus of this section. Although artichoke has a rich culinary history, recent research findings have revealed its therapeutic properties, indicating its potential to ameliorate various GI disorders and to exert antioxidant and anti-inflammatory effects. This section explores the latest research about the beneficial health effects of artichoke in modulating inflammatory responses, improving GI health, and preventing GI diseases.

A recent study conducted in human hepatocytes and adipocyte cellular models examined the anti-inflammatory effects of artichoke leaf extracts in IBD. The research investigated the health benefits of human serum enriched with metabolites derived from artichoke leaf extracts, demonstrating a protective effect against lipotoxic stress [49]. Additionally, artichoke leaf extracts were able to prevent the excessive hypertrophy and differentiation of adipocytes and exhibited chondroprotective properties in an inflammatory context. This suggests that plant-derived micronutrients, particularly polyphenols found in artichokes, known for their powerful antioxidant potential could offer a promising alternative aimed at addressing chronic diseases associated with inflammation and metabolic dysregulation [49]. Furthermore, the artichoke extracts, derived from artichoke stems, are rich in phenolic compounds, characterized by low dietary fiber and high carbohydrate content, making them a potential fat-free ingredient with significant antioxidant properties. Of particular interest is the abundance of chlorogenic acid, a type of caffeoylquinic acid known also by its antioxidant activity. Remarkably, these artichoke extracts exhibited both antioxidant and anti-inflammatory properties when tested on LPS-stimulated human THP-1 macrophages. Considering the critical role of intestinal macrophages in maintaining GI tract homeostasis, recent research has highlighted the significance of these immune cells. Disruptions in macrophage abundance and function have been associated with the disturbances in gut homeostasis, leading to chronic inflammation associated with various GI diseases [39,50,51]. Based on these findings, it is suggested that artichoke extract may indirectly contribute to GI homeostasis by influencing intestinal macrophages, implying a potential avenue for further investigation. Another study by Speciale et al. [52] has investigated the molecular mechanisms underlying the protective effects of a polyphenol-rich extract obtained from *C. cardunculus* leaves (CCLE) against acute intestinal inflammation induced by TNF-α in differentiated Caco-2 cells. 

It is well documented that IBDs are characterized by overactive mucosal immune cells and dysregulated cytokine production, leading to chronic inflammation [53]. CCLE exhibited a protective effect against the activation of the NF-κB pathway induced by TNF-α. Additionally, it was able to modulate the expression of the proinflammatory cytokine IL-8, which is stimulated by NF-κB pathway activation, and prevent the expression of COX-2 expression induced by TNF-α.

Moreover, CCLE has also demonstrated the ability to enhance cellular antioxidant defense against an altered intracellular redox status induced by TNF-α. This effect potentially involves NRF2 pathway activation. Despite being considered a waste, CCLE can be used to produce extracts rich in bioactive polyphenols that may be useful in the prevention and treatment of IBDs [52].

In the same context, another study involving an experimental colitis mouse model, reported that the treatment with CCLE was found to alleviate several symptoms associated with inflammatory bowel disease (IBD), such as diarrhea and anal edema. However, it did not result in significant weight recovery.

Moreover, in colitic mice, CCLE was shown to increase colon length, reduce in a dose-dependent manner hemoglobin in feces, and reduce alkaline phosphatase (ALP) levels. It has also considerably reduced the pro-inflammatory cytokine TNF-α. This is in favor of its anti-inflammatory effect. CCLE also appeared to protect renal function, as evidenced by the decreased urea and creatinine levels. However, it did not reverse the hepatic alterations induced by colitis. This study highlights the anti-inflammatory potential of CCLE and suggests its role as a complementary therapeutic approach for IBD management, with potential application in chronic cases and through oral administration [54].

As it was previously demonstrated, artichoke leaf extracts have also shown choleretic, hepatoprotective, bile-enhancing, antimicrobial, hypocholesterolemic, hypoglycemic, and anticancer effects both in vitro and in vivo research [5,55,56,57,58,59].

Even though artichoke by-products contain high concentrations of dietary fibers, phenolic acids, and various micronutrients, they are often discarded. Additionally, by-products extracted from artichoke stems, leaves, and bracts serve as excellent sources of pectins [60].

Given the importance of pectin in ameliorating the manifestations of IBDs, recent emerging research suggests that artichoke pectin exhibited anti-inflammatory effects in a mouse model of dextran sodium sulfate (DSS)-induced colitis [61,62]. Pectins from artichoke reduced the expression of inflammatory markers such as TNF-α, ICAM-I, IL-1β, and IL-6 in mice. This has resulted in a decrease in iNOS and TLR4 expression in favor of reducing inflammation. More interestingly, artichoke pectin increased the intestinal barrier genes expression, MUC-1 and Occludin. These findings suggest that artichoke pectin has the potential to improve IBD by inhibiting inflammation and promoting the expression of genes involved in intestinal barrier function [14].

Although the etiology of IBD is uncertain, gut microbiota dysbiosis is thought to be a pivotal factor implicated in the pathogenesis of IBD [63,64,65]. In a recent study conducted by Sasaki et al. [66], it was found that the consumption of artichoke led to alteration in gut microbiota and reduced cecal pH in mice fed on a high-fat diet (HFD). Moreover, artichoke consumption increased levels of short-chain fatty acids (SCFAs), which are vital for maintaining gut and metabolic health. Interestingly, when compared to inulin, artichoke has a distinct effect on gut microbiota, but similar effects on SCFA levels and cecal pH. 

Other bioactive components have also been shown to influence the microbiota. Specifically, the organic extract of this particular variant of artichoke was found to be predominantly responsible for altering microbiota populations. Moreover, when the organic and water-soluble extracts were combined, they collectively enhanced the synthesis of SCFA while reducing cecal pH [66].

Celiac disease (CD), a common heritable chronic inflammatory condition affecting the small intestine caused by a persistent intolerance to gluten/gliadin (prolamin), is characterized by a complex interplay between genetic and environmental factors [67,68,69,70].

In this area of investigation, Vacca et al. [71] assessed the effects of supplementing gluten-free bread with artichoke leaf extract. The study observed not only antioxidant effects, but also beneficial outcomes associated with a positive modulation of the expression of pro-inflammatory cytokines in Caco-2 cells, including TNF-α and IL1-β. This suggests the potential application of artichoke extract in developing novel gluten-free products, showing improved biological properties that prevent the occurrence of those problems associated with Celiac disease [71].

**Figure 2:**
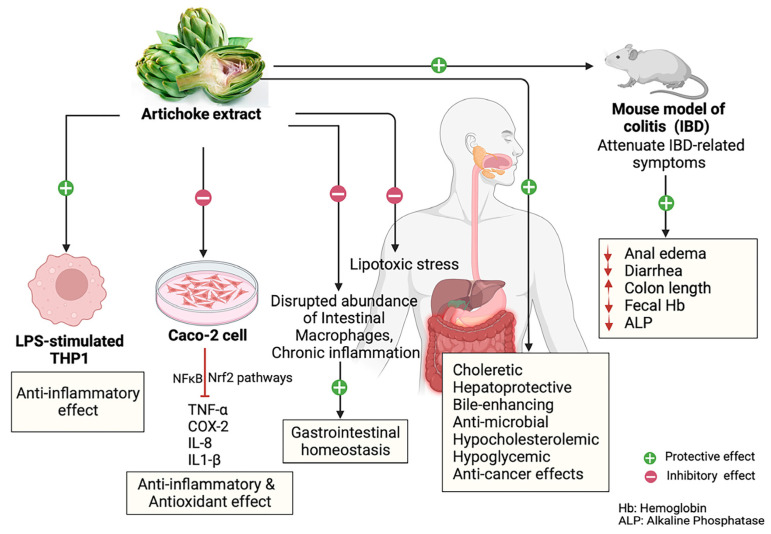
Beneficial effects of artichoke extracts on inflammatory and GI diseases; ↑ increase; ↓ decrease.

Taken together, artichoke, with its anti-inflammatory compounds and beneficial effects on GI tract physiological functions and homeostasis, presents a promising avenue to improve the health and well-being of patients diagnosed with IBDs. The growing evidence highlights the potential of artichoke and its by-products as a valuable bioactive component in the management and prevention of GI disorders associated with chronic inflammation. However, further clinical research is needed to elucidate the underlying mechanisms of action, optimal dosages, and long-term effects of artichoke-based interventions (Figure 2).

## 5. Cardiovascular System and Artichoke

Cardiovascular diseases (CVDs) represent the leading cause of mortality worldwide. In 2019, it was estimated that CVDs killed about 17.9 million people, representing 32% of all global deaths.

Out of these deaths, 85% were due to heart attack and stroke [72]. CVDs refer to a broad spectrum of diseases that can be classified using a variety of criteria. The most well-known CVDs are coronary artery disease, heart failure, myocardial infarction, cardiomyopathies, peripheral vascular diseases, hypertension, and peripheral vascular diseases. The etiology of CVDs is complicated, the risk factors which contribute to CVDs could be unmodifiable (e.g., family history, race, and age) or modifiable (e.g., hypertension, high cholesterol, obesity, type 2 diabetes) [73].

Moreover, these risk factors can trigger oxidative damage, inflammation, fibrosis, and apoptosis, which eventually aggravate the progression of CVDs. These mechanisms, in fact, may induce cardiac hypertrophy, cardiac fibrosis, endothelial dysfunction, vascular stiffness, myocardial ischemia, and, ultimately, CVDs. Therefore, targeting oxidative stress, inflammation, fibrosis, and apoptosis in hypertension, dyslipidemia, and hyperglycemia that eventually contribute to CVDs remains a primary strategy in controlling and managing the development of CVDs. As previously reported, artichoke head is well known for its nutritional values and therapeutic properties, particularly as a source of antioxidants, and can serve as an alternative treatment for various diseases. 

**Figure 3:**
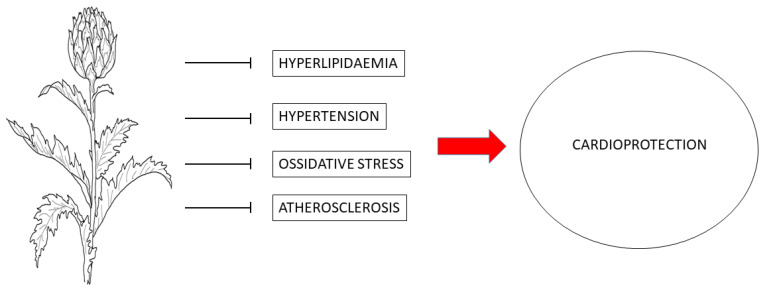
Protective mechanisms of artichoke against cardiovascular diseases.

Extensive research has consistently shown that artichoke harbors many phytochemical compounds renowned for their multifaceted health benefits. These include anti-hyperlipidemic, anti-hyperglycemic, anti-hypertensive, antioxidative, anti-inflammatory, and anti-fibrotic effects (Figure 3) [74,75,76]. The beneficial components derived from artichoke have shown important scavenging activity against reactive oxygen species (ROS) and free radicals. They act as a protective barrier, shielding biological molecules such as proteins, lipids, and DNA against oxidative damage [77].

Hyperlipidemia is characterized as an abnormally high concentration of lipids in the blood, such as triglycerides (TGs), total cholesterol (TC), and LDL-cholesterol (LDL-c) and a reduction in high-density lipoprotein cholesterol (HDL-c) values [78]. This unbalanced lipidic profile is one of the most relevant risk factors for CVD development [79].

Different studies have demonstrated that artichoke extracts influence lipid metabolism by decreasing the production of cholesterol and endogenous triglycerides by acting on their excretion or redistribution in the organism. They also enhance bile production by increasing biliary acids and cholesterol elimination with bile. Ben Salem et al. studied the effect of artichoke leaf extracts on the lipidic profile, cardiac markers, and antioxidant levels in obese rats [80]. They have found that the addition of artichoke leaf extracts (200 mg/kg and 400 mg/kg) induces in rats an improvement of the lipidic profile due to the decrease in TC, TG, and LDL-c levels and increase in HDL-c levels. Moreover, artichoke leaf extracts supplementation has also decreased the cardiac markers and increased the antioxidant enzyme SOD, GPx, and GSH activities [80].

Heidarian et al. investigated the effects of artichoke extracts on liver phosphatide phosphohydrolase and lipid profile in hyperlipidemic rats. They found that supplementing rat pellets with a mixture of 10% artichoke leaf extracts for 60 days led to a decrease in serum lipids and phosphatide phosphohydrolase activity, resulting in lower triglyceride (TG) levels [81]. In another study, individuals with primary mild hypercholesterolemia were treated for 8 weeks with artichoke leaf extracts (administered as two daily doses of 250 mg). This treatment significantly reduced total cholesterol (TC), LDL cholesterol (LDL-c), and the TC/HDL cholesterol (HDL-c) ratio, while significantly increasing HDL cholesterol (HDL-c), which plays a crucial role in preventing CVDs [82]. Additionally, to investigate the modulation of the lipidic profile by luteolin, Kwon et al. compared the effects of luteolin-enriched artichoke versus artichoke leaf on the lipidic profile. They found that artichoke leaf had a greater effect on the lipidic profile compared to luteolin-enriched artichoke [83].

CLA also induces AMP-activated protein kinase, and consequently inhibits sterol regulatory element-binding protein, resulting in a reduction in cholesterol synthesis. Moreover, CLA can induce β-oxidation and inhibit malonyl-CoA, which decrease triglyceride levels due to carnitine palmitoyl transferase stimulation [84].

Finally, inulin has been shown to modulate the lipid profile by promoting the conversion of cholesterol into bile salts, consequently reducing serum levels of very low-density lipoprotein and LDL-c [85].

Since 1930s, scientists have discovered that artichoke extract had a positive effect on atherosclerotic plaques in the arteries. Artichoke extract has been found to prevent an increase in serum cholesterol levels and the manifestations of atherosclerotic plaques in rats fed with high-fat diet (HFD). The effects of artichoke on cholesterol metabolism can be attributed to various biological activities, i.e., increasing the breakdown of cholesterol into bile salts and enhancing their elimination by stimulating bile production and flow, as well as increasing the internal production of cholesterol in the liver. Another investigation by Gebhardt and Fausel conducted on rat hepatocyte has demonstrated the inhibitory effect of artichoke extracts on cholesterol synthesis. They found a highly significant concentration-dependent inhibition of cholesterol synthesis.

The reduction in cholesterol synthesis operated by artichoke extracts persisted for several hours following the period of exposure [86].

HMG-CoA-reductase is a key enzyme in cholesterol synthesis, and HMG-CoA-reductase inhibitors generally reduce total cholesterol and LDL-cholesterol. Artichoke extract acts by inducing an indirect inhibition of the enzyme HMG-CoA-reductase, reducing the effects caused by direct inhibitors of HMG-CoA-reductase during long-term treatment. In fact, artichoke extracts effectively blocked insulin-dependent stimulation of HMG-CoA-reductase without affecting insulin in general [74,87].

The activity of artichoke leaf extracts on the regulation of the endothelial NOS (eNOS) gene also provides a protective effect. A study has explored the effects of artichoke leaf extracts on cultured human umbilical vein endothelial cells and found that it increased eNOS gene activity and expression, as well as NO production [88].

A randomized placebo-controlled trial conducted on 107 mildly hypertensive or healthy male subjects found a reduction in systolic and diastolic blood pressure after 12 weeks of oral administration of concentrate artichoke leaf juice [89].

As mentioned above, artichoke leaf extract is known for its antioxidant properties. Several studies conducted in vitro have demonstrated that the antioxidant potential of artichoke leaf extract is due to the radical scavenging and metal ion chelating effect of its constituents such as cynarin, CLA, and flavonoids [90,91].

The compounds of artichoke extracts are also involved in the prevention of development of atherosclerotic plaques. The anti-atherosclerosis effect could be the product of two mechanisms: an antioxidant effect that reduced low-density lipoprotein (LDL) oxidation and the inhibition of cholesterol synthesis [92].

## 6. Neuroprotective Effects of Artichoke

Neurodegenerative diseases such as Alzheimer’s disease (AD), Parkinson’s disease (PD), Amyotrophic lateral sclerosis, and frontotemporal lobar dementia lead to progressive damage, primarily affecting neurons. These pathologies are increasing progressively in developed societies, paralleling the aging of populations. Neurons perform essential functions such as signal transmission and information integration in the central nervous system, making them the main targets of neurodegenerative diseases [93]. 

In recent years, a new perspective is emerging, challenging the notion that neurons are the only elements essential for brain activity. The maintenance of an optimal environment for neuronal function relies on the support provided by glia cells and the proper functioning of the blood-blood barrier. This interaction between neurons and glia cells, including astrocytes, oligodendrocytes, pericytes, and microglial cells is essential for the fundamental functions of brain activity [94].

In this respect, the neurovascular unit is involved in controlling the flow of molecules that activate various processes that induce the activation of genes, which regulate the brain plasticity, neurogenesis, remodeling of dendritic spines, learning, and memory mechanisms [95].

Research in recent years has suggested that a cascade of processes collectively called neuroinflammation may partly influence the neurovascular unit contributing to neurodegeneration. Recent observations indicate that therapies targeting glial cells could provide benefits for those with neurodegenerative disorders [96].

The term “neuroinflammation” refers to the inflammatory response of central nervous system (CNS) to a neuronal insult, which is mediated by astrocytes and brain cells microglia, the main cellular mediators of the inflammatory response of the CNS. Microglia cells are the resident macrophages of the CNS, accounting for approximately 10% of the total cell population. In addition, these cells are playing a fundamental role in neurogenesis, plasticity, and neuronal regeneration. Microglia are the first line of defense against any type of brain damage, with the ability to phagocytize toxins, release cytotoxic factors, and present antigen to cells. In the absence of external stimuli, microglia are in an “inactive” state in which, due to their branched cellular morphology, they continuously monitor the neuronal microenvironment without interfering with the activity of surrounding neurons [97]. The process of microglial activation is a complex, characterized phenomenon by the acquisition of different functional phenotypes, schematically represented by M1 and M2 phenotypes, associated with neurotoxic and neuroprotective functions, respectively. In fact, once activated, the microglia undergo a morphological modification that led to take on a mobile amoeboid shape to be able to reach the site of the insult. The microglial cells can remain active for a long period, releasing cytokines and neurotoxic factors which may in turn contribute to increasing neuronal damage. The activation pattern of these cells has allowed their classification into two phenotypes: the M1 phenotype, or from classical activation, and the M2 phenotype, or from alternative activation. The M1 phenotype responds to stimulation with LPS and produces a massive response inflammatory profile with the release of IL-1β, IL-12, TNF-α, while the M2 phenotype generally presents an anti-inflammatory profile [98]. The switching from one phenotype to another is a dynamic process in which peripheral inflammation plays a decisive role.

In addition to microglia, astrocytes can release pro-inflammatory molecules in response to stimuli. Astrocytes, which are the most abundant glial cells in the central nervous system, play various roles. They are involved in the regulation of synaptic functions, in cognitive neuronal networks, supporting neuronal metabolism, maintaining the integrity of the BBB (blood-brain barrier), and regulating the cerebral blood flow [99,100].

In inflammatory reactions, astrocytes are able to communicate with microglia to amplify the immune response and activate apoptotic mechanisms that can determine neuronal death, as observed in PD [101]. As inflammatory contributors, astrocytes release pro-inflammatory cytokines, such as IL-1, TNF-α, IL-6, granulocyte colony-stimulating factor (G-CSF), granulocyte-macrophage colony-stimulating factor (GM-CSF), and macrophage colony-stimulating factor (M-CSF) in the cerebrospinal fluid [102]. However, they are also capable of producing anti-inflammatory mediators such as glutathione (GSH), ascorbic acid, glia-derived neurotrophic factor (GDNF), brain-derived neurotrophic factor (BDNF), and nerve growth factor (NGF) [103].

Similar to microglia, astrocytes can also display different phenotypes, known as the A1 and A2 phenotype. A1 astrocytes are associated with neurotoxic responses, including the phagocytosis of altered synapses and myelin debris [104], as well as the secretion of neurotoxic factors that contribute to neuronal death [104], as observed in PD. Conversely, A2 astrocytes appear to play a neuroprotective role in the brain by upregulating several neurotrophic factors, such as GDNF, which promotes neuronal survival and facilitates the repair of nervous tissue [104,105]. Therefore, the modulation and reprogramming of astrocytes and microglia could be useful in controlling neurodegenerative diseases. The available evidence strongly supports the idea that, in addition to preventing the onset of neuronal damage, nutraceuticals have the potential to reduce progressive neuronal loss by modulating inflammatory responses and decreasing levels of pro-inflammatory mediators responsible for neuronal damage.

Growing evidence highlights the neuroprotective role of polyphenols, which can exert two main actions at the cerebrovascular level. Firstly, concerning the cardiovascular system, many polyphenols exhibit anticoagulant and antiplatelet activity, thereby preventing the formation of thrombi, which are the leading causes of cerebral ischemia [106]. In addition, some of these molecules can reduce hypertension, an important risk factor for cerebral hemorrhage. The second action exerted by polyphenols is neuroprotection, which is not only through a direct action on neurons, but also on cells that modulate inflammation in the brain, such as microglia [107]. Many polyphenols can regulate gene expression without changing the DNA sequence. These molecules can modulate the action of histone deacetylase and histone acetyltransferase, enzymes involved in the control of the interaction between DNA and histones, the proteins responsible for compacting and organizing DNA within the cell nucleus. Polyphenols can reduce inflammation and increase heart and brain resistance to harmful stimuli by controlling gene expression regulatory proteins such as histone proteins and the transcriptional factor NF-κB [108].

For example, resveratrol (RSV), a well-known natural compound found in plant species belonging to the flavonoid polyphenol family [109], has been shown to have anti-inflammatory and neuroprotective properties. RSV has been identified as a promising candidate and therapeutic agent for Intracerebral Hemorrhage (IHC). Studies have shown that administering RSV following the occurrence of IHC can alleviate neuroinflammation and pro-inflammatory effects of microglia by increasing SIRT3 expression. This suggests that SIRT3 could be a potential target for RSV to inhibit inflammation [110].

It was recently reported that micronutrients have a protective effect against ischemic stroke, AD, postoperative cognitive dysfunction, and other CNS disorders [111,112,113]. An impressive body of scientific work has investigated the effect of polyphenols in pre-clinical models of stroke brain [114]. Many compounds have been shown to be effective in both cellular and animal models of stroke. An interesting aspect of this research is that many of the molecules tested promoted a beneficial effect not only when administered before the onset of stroke, but also when brain damage had already been induced. This suggests a potential use of polyphenols not only in a preventive setting, but also in a post-stroke rehabilitation phase [107]. It is widely demonstrated that oxidative stress is related to neuronal functional alterations associated with neurodegenerative diseases, including PD and AD. Nitrosamine-related compounds can cause DNA damage, oxidative stress, lipid peroxidation, and, finally, cell death.

Although limited, literature observations report that exposure to diethylnitrosamine (DEN), present in processed or preserved foods, can induce biochemical abnormalities in the brain. Some researchers have shown that a methanolic extract of exogenous artichoke leaves was able to improve the deleterious effects induced by DEN in the brain of BALB/c mice [115]. In fact, in this animal model, it was observed that the motor alterations and elevated markers of oxidative stress induced by DEN intoxication were markedly restored by treatment with high doses of artichoke extracts. From a mechanistic point of view, the artichoke significantly increased the levels of Klotho and PPARγ, factors with neuroprotective effects, in the brain tissue of mice exposed to DEN. A significant reduction in caspase-3 and Bax levels was also observed, while an increase in Bcl-2 expression was observed following treatment with artichoke. These results led to the conclusion that artichoke exerted neuroprotective effects against DEN-induced brain toxicity through antioxidant and anti-apoptotic action [115].

In recent years, there is growing evidence that polyphenols derived from plants have beneficial effects, and therefore they can be exploited for food purposes for their biological properties, using them as a unique nutraceutical for the supplementary treatment of metabolic and/or inflammatory disorders [116]. In particular, the artichoke leaf extract has several beneficial properties for health. Some studies have found that it has both anti-inflammatory, antioxidant and anti-bacterial effects, as well as the ability to inhibit cholesterol biosynthesis and the oxidation of low-density lipo-proteins [108]. These effects have been attributed to the presence of polyphenolic compounds contained in the artichoke.

In accordance with these findings, it was recently found in a mouse model of induced obesity that the ethanolic extract of artichoke extracts has beneficial effects on neuroinflammatory parameters [117].

It is well-documented that obesity can cause neuroinflammation and increased oxidative stress in the brain [118]. In the same context, Piccinini et al. explored the effect of artichoke leaf ethanol extract on inflammatory and oxidative stress in different brain areas, including the hypothalamus, prefrontal cortex, hippocampus, striatum, and the cerebral cortex, in mice fed with high-fat diet [117].

This study reveals that the ethanolic extract of artichoke leaves conferred benefits to the brains of obese mice by reducing the production of inflammatory cytokines. These findings corroborate previous studies, suggesting that the leaf ethanolic extract of artichoke may have therapeutic potential for treating obesity, alongside its recognized anti-inflammatory and antioxidant properties [118].

According to Santos et al., the polyphenolic content of the artichoke, in addition to phytosterols, may have a protective effect due to their ability to contain oxidative and inhibit inflammatory and antioxidant inflammatory pathways [84]. Another recent study by Guo et al. has demonstrated in an in vitro model of encephalitis that CLA can significantly increase cell survival, decrease TLR2, TLR9, and MyD88 levels, and inhibits NF-κB activation. These findings suggest that CLA interferes with the signaling pathways involved in inflammatory responses [118].

Polyphenols have been shown to improve mood by increasing serotonin levels in the brain, stimulating the production of brain-derived neurotrophic factor (BDNF), and reducing inflammation [119]. In addition to causing apoptosis and neuronal damage, oxidative stress is a key factor in neuroinflammation, anxiety, and depression [120,121,122].

Isochlorogenic acid B (ICAB), also known as 3,4-dicaffeoylquinic acid, is a dietary flavonoid present in various plants including artichoke. It possesses multiple pharmacological effects, such as antioxidative, anti-inflammatory, and hepatoprotective effects [117,123,124,125].

Also, the flavonoid ICAB, present in the artichoke, has shown potential neuroprotective properties. In a mouse model of lead (Pb)-induced neurotoxicity, it was demonstrated that ICAB treatment suppressed the activation of TLR4, MyD88, GSK3β, p38, and NF-κB, resulting in a significant reduction in the inflammatory cytokine levels, such as TNF-α and IL-6. These results suggest that the integration of this artichoke derivative can reduce the Pb-induced inflammatory response and depression through the TLR4/MyD88/GSK3β/p38 signaling pathway [126].

Furthermore, ICAB increases BDNF expression, a factor capable of providing neuroprotection against anxiety, depression, and memory impairment by increasing phosphorylation of the transcription factor CREB, which is involved in the expression of numerous proteins related to neurogenesis [127,128].

Chronic oxidative stress may represent a cause for the development of neurodegenerative diseases and free radicals may act as secondary messengers capable of modifying inflammatory responses by microglia, altering kinase cascades and activating transcription factors, such as NF-κB [129,130].

It has been shown in several studies that the modulation of an excessive inflammatory response by activated microglial cells may attenuate the severity of neurodegenerative diseases [131,132]. Indeed, it has been reported that artichoke extracts show neuroprotective effects in a mouse model of AD. The neuroprotective effects of artichoke leaf extracts were recently studied in a murine model of sporadic AD induced by streptozotocin, in a preparation made of solid lipid nanoparticles. In this study, in fact, was shown a significant improvement in cognitive functions and recovery of spatial memory, as well as a marked reduction in the inflammatory biomarker TNF-α. Furthermore, reduced levels of both β-amyloid and tau protein were observed. These results suggest the strong potential of artichoke bracts as a herbal medicine, opening the use of agro-industrial waste to future medicinal prospects [133].

Recently, a study investigated the attenuation of adverse effects caused by aflatoxin (AFBA1) in the brains of B1 rats. AFBA1 is known to increase lipid peroxidation, leading to cytotoxic damage in the brain, and contributing to neurodegenerative diseases. The study examined the effects of artichoke leaf extract in male rats following 42 days of exposure to AFBA1, assessing the histopathological architecture of the brain. Furthermore, brain-specific plasma markers, lipid profile, and antioxidant enzyme activities were evaluated, along with free radicals to assess the neuroprotective potential of artichoke [41]. Interestingly, it was observed that the treatment of rats with artichoke leaf extract resulted in a significant reduction in microglial infiltration, TNF-α levels, and free radical production. This highlights the neuroprotective effects in the in vivo model of neurodegeneration.

A study by Mekkey et al. investigated the anti-inflammatory, antioxidant, and anti-apoptotic effects of the whole artichoke dry extract in a mouse model of PD. This study revealed beneficial effects against PD experimentally induced by rotenone, highlighting anti-inflammatory and anti-apoptotic properties in addition to its ability to reduce the expression of alpha synuclein, a pathological hallmark of PD [134].

Artichoke extract has been observed to improve brain damage and memory deficits through inhibition of oxidation and inflammation. This research demonstrated that the artichoke can be a promising alternative therapeutic agent for the treatment of neurological diseases [115,133]. The beneficial and medicinal properties of the artichoke are due to its bioactive phenolic compounds, in particular the caffeoylquinic acid derivative cynarin [16]. In this regard, previous studies have highlighted that the increase in glutamate actively contributes to the neuronal damage observed in neurodegenerative diseases. In fact, the attenuation of glutamate neurotransmission and the reduction in the synaptic release of this neurotransmitter leads to effective neuroprotection [123].

A study conducted on the nerve terminals of the rat cerebral cortex (synaptosomes) has allowed for understanding the molecular mechanisms underlying the regulation of the availability of synaptic vesicles and glutamate exocytosis. The study demonstrated that cynarin acts on the PKA signaling pathway in regulating glutamate release, decreasing Ca^2+^ influx through P/Q-type Ca^2+^ channels [135]. This result demonstrates that cynarin inhibits glutamate release in rat cerebral synaptosomes and provides an interesting contribution to understanding the neuroprotective effect of artichoke in the brain. This suggests that cynarin could be potentially used for the treatment of brain diseases associated with glutamate excess.

**Figure 4:**
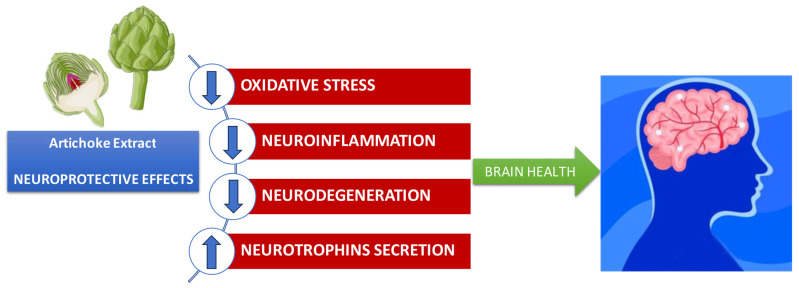
Beneficial effects of artichoke extract in neurodegenerative diseases; ↑ increase, ↓ decrease.

From what has been illustrated, it emerges that artichoke leaf extracts can represent a valid and promising botanical therapeutic approach for the treatment of neuroinflammation and several pathologies associated with inflammation, including neurodegenerative disorders (Figure 4). The phytochemical analysis of artichoke bract extract reveals a high concentration of caffeoylquinic acid, apigenin, luteolin, and quercetin, highlighting the richness of the total content of phenols and flavonoids. The use of nano formulations, such as solid lipid nanoparticles, could result in greater effectiveness of the preparations in overcoming problems of bioavailability and/or degradation of the living organism, as reported in a previous study [133].

In any case, evidence of significant improvements in memory, cognitive functions, motor skills, and a significant reduction in inflammatory levels, histopathological, and functional signs that differentiate various neurodegenerative pathologies, such as AD and PD, supports the efficacy of these compounds.

Further research is needed to propose a botanical treatment for neurodegenerative diseases or chronic inflammatory pathologies, as well as future therapeutic perspectives in the therapeutic use of agro-industrial waste such as artichoke bracts.

## 7. Pharmacological Use of Artichoke Extracts

Several studies have revealed that *C. Scolymus* is available in a pharmaceutical form and as a functional food supplement.

Though there have been few clinical studies on the topic, the antioxidant effects of artichokes have been investigated in a number of in vitro and animal models. 

Patient data from digestive or cardiovascular disorders are presented in the majority of clinical studies. Artichoke leaf extract has been used traditionally to prevent or treat hepatobiliary and digestive disorders [80]. 

In this respect, a study aimed to assess the therapeutic effects of *C. scolymus* on liver function and hemodynamic parameters in patients with nonalcoholic steatohepatitis (NASH) was conducted by Rangboo and coll [136]. After two months of administrating *C. scolymus* extract (6 tablets daily containing 2700 mg of extract), a notable improvement was observed in patients. Indeed, patients showed significant reduction in the average weight, as well as in serum levels of alanine and aspartate transaminase (both recognized as effective biomarkers for diagnosing hepatic damage), blood sugar, total cholesterol, LDL, and triglycerides. Additionally, a significant decrease in systolic blood pressure was reported. These findings suggest the potential hepatoprotective and hypolipidemic effects of *C. scolymus* in managing NASH.

Moreover, the use of plant-derived compounds in complementary and alternative medicine for individuals with metabolic disorders plays a crucial role in preventing cardiovascular diseases. In this regard, the literature emphasizes the substantial utilization of artichoke. 

Interestingly, Rezazadeh et al. have found that artichoke leaf extract supplementation at a dose of 1800 mg/day for 12 weeks exhibits remarkable antioxidant potential in 80 women diagnosed with metabolic syndrome. This extract significantly decreased the ox-LDL level [137].

Building on this, Rondanelli et al. have investigated the hypoglycemic effect of *C. scolymus* extracts attributing its efficacy to the presence of chlorogenic acid, which is a potent inhibitor of glucose 6-phosphate translocase and dicaffeoylquinic acid derivatives known to modulate the activity of alpha-glucosidase. 

This study, involving impaired fasting glucose patients, implemented a dietary treatment comprising two daily oral doses (before lunch and dinner) of 500 mg *C. scolymus* tablets (containing 500 mg of artichoke extract) over 8 weeks. The outcomes obtained confirmed that *C. scolymus* supplementation significantly influences metabolic parameters in impaired fasting glucose patients [138]. 

Results from a study conducted by Ardalani et al. reported that the consumption of *C. scolymus* as powder in capsule form (500 mg twice daily) for 8 weeks ameliorates the body mass index and reduces the systolic blood pressure in hypertensive patients [139].

Further investigations suggested that artichoke supplementation may potentially reduce both systolic and diastolic blood pressure in hypertensive patients. Notably, supplementation with (tablet of dry extract of *C. scolymus*; 50 and 100 mg of artichoke juice concentrate) for 8–12 weeks has shown significant improvements in diastolic blood pressure [89,140,141].

In addition, dietary supplementation with artichoke seems to positively modulate endothelial function in hypercholesterolemia. In fact, the administration of artichoke leaf extract as juice (20 mL/die of frozen artichoke juice dissolved in water with a few drops of lemon juice) can modulate endothelium in vivo, thus regulating endothelial function, as evidenced in a study conducted on hyperlipemic patients [141].

A study conducted on patients with functional dyspepsia, aimed to assess the effectiveness of artichoke leaf extract, reported that the administration of two capsules from a commercially available preparation (containing 320 mg of artichoke leaf extract) was able to alleviate symptoms and improve the quality of life in patients with functional dyspepsia [142].

Another study by Rezazadeh et al. explored the potential antioxidant effects of 12 weeks of supplementation with artichoke leaf extract on patients with metabolic syndrome, a condition known to increase the risk of developing type 2 diabetes and cardiovascular diseases [143]. The participants, who took a daily hydro-alcoholic artichoke leaf extract supplement totalling 1800 mg, distributed in four tablet administrations (one before breakfast, one before dinner, and two before lunch), exhibited a significant reduction in oxidized-LDL and triglyceride serum levels at the conclusion of the treatment period [143].

Nevertheless, it is important to acknowledge that many clinical studies concerning the use of artichoke in neurodegenerative contexts have certain limitations. These include relatively short intervention periods, a high number of administered tablets, and the fact that the precise mechanisms of action have not been fully elucidated. 

## 8. Conclusions

Artichoke, a plant commonly used in the Mediterranean diet and traditional medicine, contains several bioactive compounds. Various studies have demonstrated its potential as an anti-inflammatory, lipid-lowering, antimicrobial, and neuroprotective agent due to its phytochemical composition. With the rapid increase in the world population and depletion of resources, there is a growing need for more sustainable and efficient use of natural resources. Understanding the correct industrial exploitation remains important for promoting complete and adequate recycling of plant crops. 

The development of new antioxidants presents a safe and effective way to strengthen the body’s defense system against potentially harmful molecules, such as free radicals and inflammatory mediators.

The observations reported in this review indicate that compounds extracted from artichoke could facilitate the protection against various inflammatory diseases, thus suggesting their use as a dietary supplement in combination with conventional therapies. In conclusion, this review could represent a useful starting point to focus the research on compounds derived from artichoke for potential incorporation into possible ingredients for functional foods and nutraceuticals. These products could have applications in preventive medicine and the treatment of neurodegenerative diseases.

## Figures and Tables

**Table 1:** Summary of the principal mechanisms induced by artichoke chemical compounds.

Major Group	Chemical Compound	Action Mechanisms [Reference]
Flavonoids	Luteolin	Antioxidant action [16] Lipid profile modulation [29] Anticholestatic action [30] Cholerectic action [30] Antimicrobial action [31]
Flavonoids	Luteolin 7-O-glucoside	Antioxidant action [32] Hepato-protective action [33] Anticholestatic action [30] Cholerectic action [33]
Flavonoids	Luteolin 7-O-rutinoside	Antioxidant action [6] Anti-hyperlipidemic [34]
Flavonoids	Luteolin 7-O-glucuronide	Anti-inflammatory action [35]
Flavonoids	Apigenin Apigenin7-O-rutinoside Apigenin 7-O-glucoside Apigenin 7-O-glucuronide	Antioxidant action [6] Antimicrobial action [33]
Hydroxycinammic acids	Caffeic acid	Antioxidant action [6,16] Antimicrobial action [6,16] Hepato-protective action [34] Anticholestatic action [34] Cholerectic action [34]
Hydroxycinammic acids	Ferulic acid	Antimicrobial action [16]
Mono-caffeoylquinic acids	3-O-caffeoylquinic acid 4-O-caffeoylquinic acid 5-O-caffeoylquinic acid	Anti-inflammatory action [36] Antimicrobial action [37]
Di-caffeoylquinic acids	1,3-di-CQA	Hepato-protective action [5] Anti-inflammatory action [38,39]
Di-caffeoylquinic acids	1,5-di-CQA	Antiglycative action [40]
Di-caffeoylquinic acids	3,4-di-CQA 3,5-di-CQA 4,5-di-CQA	Antioxidant action [41]
Sesquiterpene lactones	Cynaropicrin	Anti-inflammatory action [19] Antiparasitic action [20] Anti-tumor action [23,26] Antioxidant action [25] Neuroprotective action [25]
Sesquiterpene lactones	Dehydrocynaropicrin Grosheimin Cynaratriol 8-deoxy-11,13-dihydroxygrosheimin	Anti-inflammatory action [27]
